# A Case of Rheumatoid Arthritis and Limited Systemic Sclerosis Overlap Successfully Treated with Tocilizumab for Arthritis and Concomitant Generalized Lymphadenopathy and Primary Biliary Cirrhosis

**DOI:** 10.1155/2014/386328

**Published:** 2014-04-15

**Authors:** Eiko Saito, Shinji Sato, Shinichi Nogi, Noriko Sasaki, Naofumi Chinen, Kiri Honda, Takayuki Wakabayashi, Chiho Yamada, Naoya Nakamura, Yasuo Suzuki

**Affiliations:** ^1^Division of Rheumatology, Department of Internal Medicine, Tokai University School of Medicine, 142 Shimokasuya, Isehara 160-8582, Japan; ^2^Department of Pathology, Tokai University School of Medicine, 142 Shimokasuya, Isehara 160-8582, Japan

## Abstract

A 57-year-old woman with rheumatoid arthritis (RA) and limited systemic sclerosis (lSSc) was suspected to have lymphadenopathy and primary biliary cirrhosis (PBC). Lymph node biopsy showed reactive follicular lymphadenopathy with intrafollicular plasmacyte infiltration that was interleukin-6 positive by immunohistostaining. Because of gradually worsening arthritis, tocilizumab was administered and arthritis improved markedly. Interestingly, lymphadenopathy and PBC improved simultaneously. This suggested that interleukin-6 might play an important role in reactive lymphadenopathy and PBC associated with RA/lSSc.

## 1. Introduction

More than 50% of patients with rheumatoid arthritis (RA) exhibit lymphadenopathy [1], and reactive follicular hyperplasia is one of most distinctive histological characteristics of it [2]. There are 3 main causes of lymphadenopathy complicated with RA. These are due to extra-articular manifestation of RA itself, complication of Sjögren syndrome [3], and treatment with immunosuppressive agents including methotrexate (MTX) or tumor necrosis factor-*α* (TNF-*α*) blockers [4–9].

If a patient develops lymphadenopathy during treatment with MTX, the possibility of MTX associated lymphoproliferative disease (LPD) arises and MTX should be discontinued promptly [8]. Here we report a case of RA overlapping with limited systemic sclerosis (lSSc) with refractory arthritis, generalized lymphadenopathy, and primary biliary cirrhosis (PBC). In this case, treatment with tocilizumab (TCZ) was successful in management of these concurrent conditions.

## 2. Case Report 

**Figure 1:**
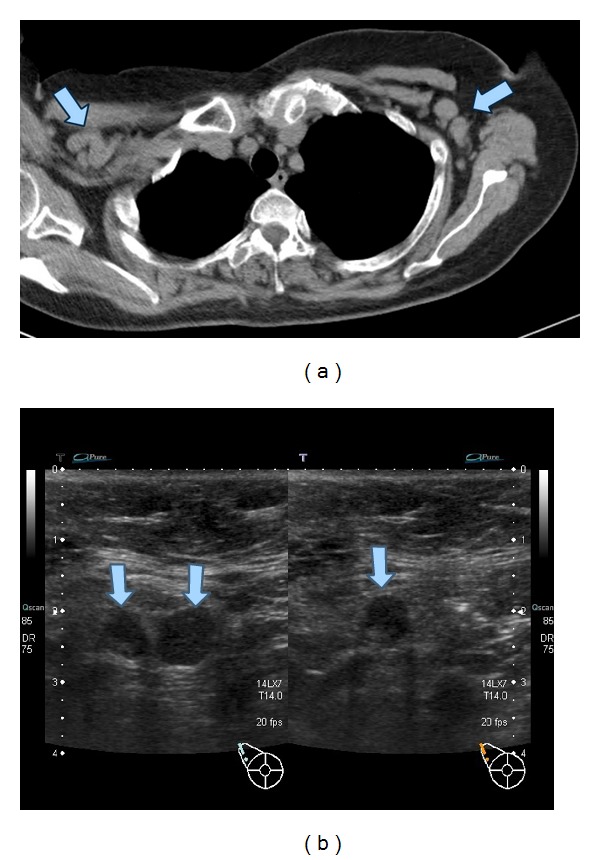
Lymphadenopathy at first admission. Lymphadenopathy was seen in both axial lymph nodes at first admission (a). Malignant lymphoma was suspected because lymph nodes echogram showed elliptical shape and reticular or hypoechoic pattern with deviation of lymph node hilum (b).

**Figure 2:**
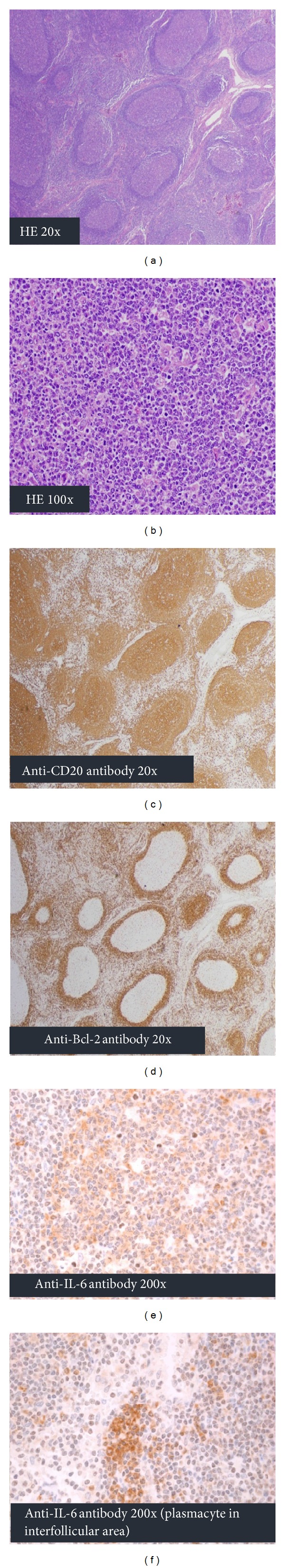
Histopathological and histoimmunochemical features of lymph node biopsy. Lymph node biopsy showed follicular hyperplasia and germinal center proliferation with narrowed mantle-zone ((a): HE stain, ×20). No dysplasia was seen in germinal center and hemophagocytic cells are scattered in this area ((b): HE stain, ×100). The lymphocytes forming in the germinal center were CD20 positive (c), BCL-2 negative (d), and IL-6 positive (e). Plasmacytes in interfollicular area were also IL-6 positive (f).

**Figure 3:**
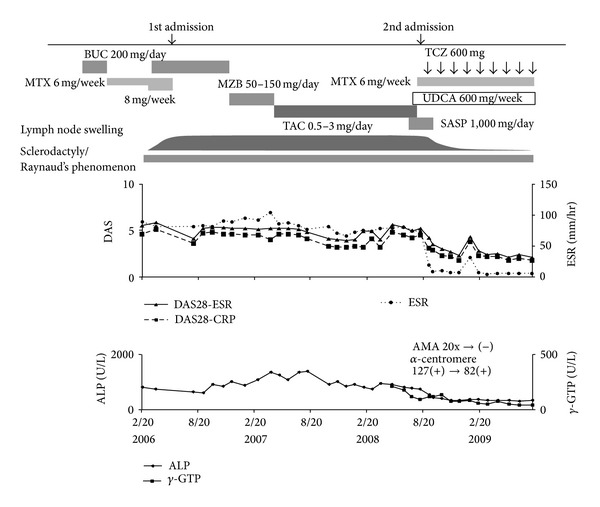
Clinical course and changes in articular manifestations, lymph node swelling, and serum ALP, *γ*-GTP concentration. The improvements of articular manifestations, lymph node swelling, and serum ALP, *γ*-GTP concentration were closely linked to TCZ therapy.

A 57-year-old woman with RA was referred to Tokai University Hospital for further evaluation and treatment. She was initially treated with 200 mg daily of Bucillamine without improvement. Therefore, 6 mg weekly of MTX was started and then increased to 8 mg weekly and continued for another 8 weeks. At that time, her joint symptoms were still active and she had polyarthritis. Although she had Raynaud's phenomenon and sclerodactyly, she had no keratoconjunctivitis sicca and xerostomia, and parotid gland swelling was not observed. Therefore, she was diagnosed with lSSc but did not fulfill the criteria of Sjögren syndrome. Laboratory findings revealed an erythrocyte sedimentation rate (ESR) of 89 mm/hr, C-reactive protein (CRP) of 3.85 mg/dL. Anti-CCP antibody and RF were positive. Anti-nuclear antibody (centromere pattern) and anti-centromere antibody were also positive. She had 6 tender joints (TNJ) and 8 swollen joints (SWJ). DAS28-ESR was 5.83 which indicated that she had still high disease activity. Therefore, she was admitted to our hospital to introduce infliximab (IFX) therapy. Prior to starting the combination therapy with IFX and MTX, computed tomography (CT) was performed to rule out active tuberculosis infection. CT revealed that she had generalized lymphadenopathy (Figure 1(a)) and she was suspected to have malignant lymphoma (ML) based on the results of ultrasound sonography (Figure 1(b)). Treatment with IFX and MTX was discontinued in case generalized lymphadenopathy was due to MTX. A lymph node biopsy was immediately performed from the swollen right neck lymph node. Histology showed follicular hyperplasia throughout the lymph node and germinal center proliferation with a narrowed mantle-zone (Figure 2(a)). Germinal centers consisted of mainly centroblasts and centrocytes with tingible body macrophages, indicating reactive follicular hyperplasia (Figure 2(b)). These centroblasts and centrocytes showed CD20 positive, BCL-2 negative and IL-6 positive immunohistochemically (Figures 2(c)–2(e)). In interfollicular area, many mature plasma cells are infiltrated and these also showed IL-6 positive (Figure 2(f)). Epstein-Bar virus in situ hybridization was negative (not shown). At the same time, alkaline phosphatase (ALP) and gamma glutamyl transpeptidase (*γ*-GTP) gradually increased. Blood test revealed positive anti-mitochondrial antibody (AMA) and otherwise unexplained high ALP and *γ*-GTP concentrations that lead to the diagnosis of PBC. Thus, treatment with ursodeoxycholic acid was initiated. In spite of discontinuation of MTX, lymphadenopathy did not improve, contrary to expectations. Because her joint symptoms worsened rapidly during the same period, mizoribine, tacrolimus, and sulfasalazine were used to relief her articular inflammation in turn; however, none of them were effective. Her lymphadenopathy persisted and the size of lymph nodes remained the same during the clinical course. Because her arthritis worsened gradually, she was again hospitalized to receive combination therapy with tocilizumab (TCZ), a humanized monoclonal antibody against interleukin-6 (IL-6) receptor, and MTX. On the second admission, TNJ count was 3 and SWJ count was 5. Generalized lymph node swelling was still observed. Laboratory data indicated an ESR of 75 mm/hr, CRP of 4.21 mg/dL, and matrix metalloproteinase-3 of 739 ng/mL. DAS28-ESR score of 5.39 showed she still had high disease activity. ALP and *γ*-GTP were elevated to 787 U/L and 117 U/L, respectively. Treatment with 600 mg of TCZ monthly plus 6 mg of MTX weekly was initiated (Figure 3). After introduction of TCZ, her polyarthritis improved markedly.

**Figure 4:**
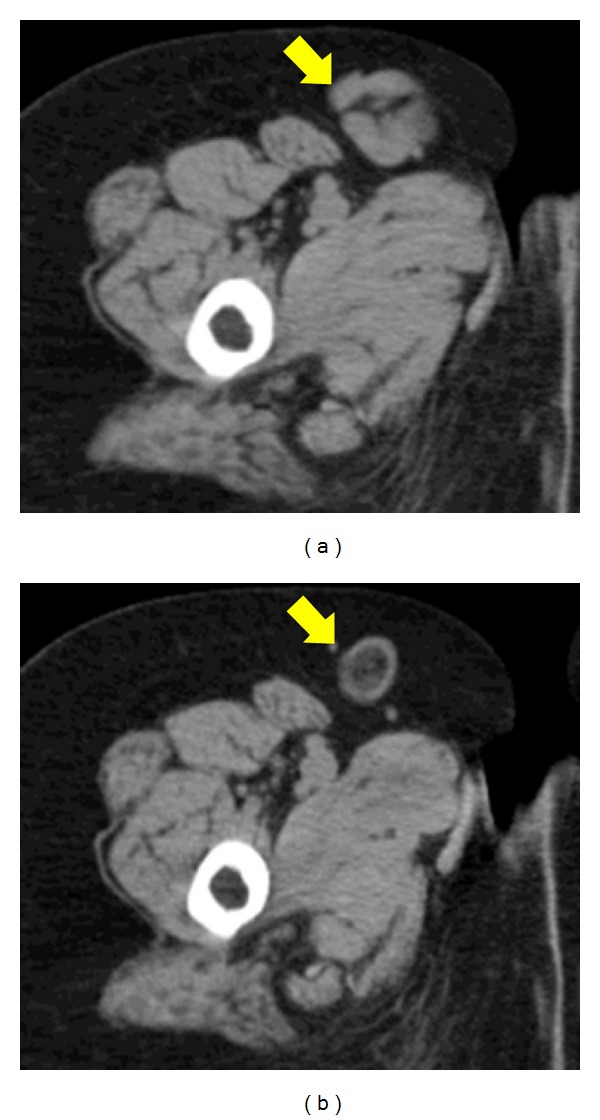
Change in lymph node size by TCZ therapy. Generalized lymphadenopathy was observed before TCZ therapy (left panel, right inguinal area) but it regressed rapidly with central necrosis after TCZ therapy (right panel, right inguinal area).

Nine months after the introduction of the TCZ, her DAS28-ESR score was 1.53 (her TNJ count was 1 and SWJ count was 2) showing clinical improvement. Interestingly, the generalized lymphadenopathy regressed rapidly (Figure 4) and serum ALP and *γ*-GTP concentration as well as AMA titer also decreased to within normal range in parallel with the improvement of polyarthritis. However, sclerodactyly and Raynaud's phenomenon did not improve apparently even after the initiation of TCZ therapy. Her arthritis has been well controlled for more than 2 years and she continues to take TCZ and MTX with no adverse events or complications.

## 3. Discussion

Here we reported a patient with overlapping RA and lSSc who was successfully treated with TCZ for polyarthritis as well as generalized lymphadenopathy and PBC. The predominant feature of present case is the efficacy of TCZ for generalized lymphadenopathy and PBC.

Lymphadenopathy is one of the extra-articular manifestations of RA and increasing relative risk of ML in RA and Sjögren syndrome is also well known [3]. Besides, it has been reported that MTX and TNF-*α* blockers induce lymphadenopathy in RA that are known as “iatrogenic immunodeficiency-associated lymphoproliferative disorders” [10]. Histopathological patterns of lymphadenopathy associated with RA are diverse. According to Kojima et al. [11], LPDs in RA are classified as benign, atypical, and malignant types. Histopathological finding of lymphadenopathy that is frequently seen in RA is reactive follicular hyperplasia with interfollicular plasmacytosis, which is classified as benign [2].

As the pathological manifestation of the present case showed reactive follicular hyperplasia and appeared to be benign, we suspected that a generalized lymphadenopathy was due to MTX associated LPD because it was discovered during MTX therapy. Therefore, we discontinued the administration of MTX immediately. However, lymphadenopathy did not improve in spite of MTX discontinuation. TNF-*α* blockers should be avoided in such a condition because Food and Drug Administration has issued warning that there is an increased risk of malignancy in patients treated with TNF-*α* blockers [1]. Numata et al. reported an RA patient with lymphadenopathy that showed follicular hyperplasia with increased expression of IL-6 in germinal centers that was identical to the pathologic characteristics of Castleman disease and the lymphocytes from this lymph node produced IL-6 in in vitro culture [12]. This patient showed the same histopathological features and also exhibited the increased expression of IL-6 in the germinal centers and interfollicular area. These results might indicate that one of histopathological characteristics of RA-associated lymphadenopathy are the same as those of Castleman disease. Therefore, TCZ is a possible candidate to treat RA patients with these conditions.

In addition, this case was complicated with PBC and showed high serum ALP and *γ*-GTP levels. However, these values decreased after injection of TCZ and AMA also became negative in parallel with the normalization of serum ALP and *γ*-GTP level after 9 months of TCZ treatment. These findings suggest that increased serum IL-6 concentration might play a role in PBC. However, as far as we know, there are few reports of the association of PBC with increased serum IL-6 level. Yamashiki et al. reported that sera from PBC patients showed a higher percentage of IL-6 receptor positive T lymphocytes by PHA activation compared with healthy controls [13]. They also reported that there is a significant correlation between the percentage of IL-6 positive T lymphocytes and AMA titer [13]. These results suggest that IL-6 might play an important role in pathogenesis of PBC. Improvement of PBC in this case might support this hypothesis. However, this is only a single case and further study of similar patients should help draw a more rigorous conclusion.

Currently, TCZ, monoclonal anti-IL-6 receptor antibody, has been approved for treatment of RA, Castleman disease, and juvenile idiopathic arthritis in Japan. Moreover, TCZ is expected to be potentially useful in the treatment of other IL-6 associated diseases such as Crohn's disease. In this context the present case might suggest that the potential use for the treatment of LPD and PBC associated with RA/lSSc overlap. Although Shima et al. reported that sclerosis of skin was improved by TCZ [14], sclerodactyly and Raynaud's phenomenon did not improve obviously in this patient. As the stage of her skin thickening was late phase and involved in only the fingers, it might be difficult to evaluate the improvement of skin thickening.

In summary, we reported a case of refractory RA/lSSc overlapping with generalized lymphadenopathy and PBC successfully treated with TCZ. This case suggested that increased serum IL-6 concentration might play an important role in lymphadenopathy and PBC associated with RA/lSSc. TCZ might be effective for these conditions as well as for treatment of the articular manifestation in RA.

## References

[1] Robertson MD, Hart FD, White WF, Nuki G, Boardman PL (1968). Rheumatoid lymphadenopathy. *Annals of the Rheumatic Diseases*.

[2] Kojima M, Hosomura Y, Itoh H (1990). Reactive proliferative lesions in lymph nodes from rheumatoid arthritis patients: a clinicopathological and immunohistological study. *Acta Pathologica Japonica*.

[3] Theander E, Manthorpe R, Jacobsson LTH (2004). Mortality and causes of death in primary Sjögren’s syndrome: a prospective cohort study. *Arthritis and Rheumatism*.

[4] Georgescu L, Quinn GC, Schwartzman S, Paget SA (1997). Lymphoma in patients with rheumatoid arthritis: association with the disease state or methotrexate treatment. *Seminars in Arthritis and Rheumatism*.

[5] Moseley AC, Lindsley HB, Skikne BS, Tawfik O (2000). Reversible methotrexate associated lymphoproliferative disease evolving into Hodgkin’s disease. *Journal of Rheumatology*.

[6] Askling J, Fored CM, Baecklund E (2005). Haematopoietic malignancies in rheumatoid arthritis: lymphoma risk and characteristics after exposure to tumour necrosis factor antagonists. *Annals of the Rheumatic Diseases*.

[7] Wolfe F, Michaud K (2007). The effect of methotrexate and anti-tumor necrosis factor therapy on the risk of lymphoma in rheumatoid arthritis in 19,562 patients during 89,710 person-years of observation. *Arthritis and Rheumatism*.

[8] Rizzi R, Curci P, Delia M (2009). Spontaneous remission of “methotrexate-associated lymphoproliferative disorders” after discontinuation of immunosuppressive treatment for autoimmune disease. Review of the literature. *Medical Oncology*.

[9] Wong AK, Kerkoutian S, Said J, Rashidi H, Pullarkat ST (2012). Risk of lymphoma in patients receiving antitumor necrosis factor therapy: a meta-analysis of published randomized controlled studies. *Clinical Rheumatology*.

[10] Gaulard P, Swerdlow SH, Harris NL, Jaffe ES, Sundstrom C, Swerdlow SH, Campo E, Harris NL (2008). Other iatrogenic immunodeficiency-associated lymphoproliferative disorders. *World Health Organization Classification of Tumours. Pathology and Genetics: Tumours of Haematopoietic and Lymphoid Tissues*.

[11] Kojima M, Motoori T, Nakamura S (2006). Benign, atypical and malignant lymphoproliferative disorders in rheumatoid arthritis patients. *Biomedicine and Pharmacotherapy*.

[12] Numata Y, Matsuura Y, Onishi S (1991). Case report: interleukin-6 positive follicular hyperplasia in the lymph node of a patient with rheumatoid arthritis. *The American Journal of Hematology*.

[13] Yamashiki M, Kosaka Y, Nishioka J (1998). Flow cytometric analysis of IL-6 Receptors on peripheral lymphocytes in patients with primary billiary cirrhosis. *Journal of Clinical Laboratory Analysis*.

[14] Shima Y, Kuwahara Y, Murota H (2010). The skin of patients with systemic sclerosis softened during the treatment with anti-IL-6 receptor antibody tocilizumab. *Rheumatology*.

